# Leveraging manifold learning techniques to explore white matter anomalies: An application of the TractLearn pipeline in epilepsy

**DOI:** 10.1016/j.nicl.2022.103209

**Published:** 2022-09-22

**Authors:** E. Roger, A. Attyé, F. Renard, M. Baciu

**Affiliations:** a Univ. Grenoble Alpes, CNRS LPNC UMR 5105, Grenoble, France; b Neuroradiology and MRI, Grenoble Alpes University Hospital, Grenoble, France; c GeodAIsics, Grenoble, France

**Keywords:** White matter, Tractography, Epilepsy, Manifold learning, Precision medicine, Neurocognition

## Abstract

An accurate description of brain white matter anatomy *in vivo* remains a challenge. However, technical progress allows us to analyze structural variations in an increasingly sophisticated way. Current methods of processing diffusion MRI data now make it possible to correct some limiting biases. In addition, the development of statistical learning algorithms offers the opportunity to analyze the data from a new perspective. We applied newly developed tractography models to extract quantitative white matter parameters in a group of patients with chronic temporal lobe epilepsy. Furthermore, we implemented a statistical learning workflow optimized for the MRI diffusion data – the TractLearn pipeline – to model inter-individual variability and predict structural changes in patients. Finally, we interpreted white matter abnormalities in the context of several other parameters reflecting clinical status, as well as neuronal and cognitive functioning for these patients. Overall, we show the relevance of such a diffusion data processing pipeline for the evaluation of clinical populations. The “global to fine scale” funnel statistical approach proposed in this study also contributes to the understanding of neuroplasticity mechanisms involved in refractory epilepsy, thus enriching previous findings.

## Highlights

• We performed a global-to-specific white matter (WM) analysis in patients with temporal lobe epilepsy (TLE)
• Track-weighted fractional anisotropy was related to patients' clinical and cognitive status
• We used TractLearn, a manifold learning pipeline, to automatically detect patient-specific WM lesions
• We found WM abnormalities in several fascicles, clustered on certain tract segments
• Our analyses highlighted interindividual variability and the need for precision medicine

## 1 Introduction

Neurological disorders associated with white matter (WM) lesions include greater cognitive and behavioral deficits compared to isolated cortical damage, and WM impairments are often a sign of a worse prognosis for recovery (e.g., Duffau et al., 2014, Kiran and Thompson, 2019, Lunven et al., 2015, Sidaros et al., 2008). Typically, neurosurgeons pay particular attention to preserving the connections of the major white matter networks, as the WM macrostructure is little subject to inter-individual variability and reorganization (Duffau, 2017, Ius et al., 2011). However, there is some neuro-adaptability of the WM microstructure. Several studies have indeed demonstrated that the density of myelinated axons or that the degree of myelination itself can vary depending on several factors and interactions (Walhovd et al., 2014). While structural connectivity supports functional wiring, the function also directly modulates certain properties of structural connections for example (Hervé et al., 2013). The WM microstructure has thus emerged as an important feature to consider when studying neuroplasticity, but an accurate quantification of its variations in neurotypical or neuropathological populations remains an ongoing challenge (Yang et al., 2021).

Temporal lobe epilepsy (TLE) is characterized by seizures arising from a dysfunctional temporal lobe epileptogenic region, typically located within temporal medial structures (Burianová et al., 2017). Changes in structural and functional networks in this form of epilepsy, which is particularly resistant to antiepileptic drugs, are based on complex intertwined neuroplasticity mechanisms (e.g., Englot et al., 2016, Roger et al., 2019a, Roger et al., 2019b). Given the significant role of lateral and medial temporal regions in cognitive functions such as language and memory, recurrent seizures would modify in time the function and the structure of these high-level functional networks (Balter et al., 2019, Banjac et al., 2021a, Banjac et al., 2021b, Berg and Scheffer, 2011, Roger et al., 2019a, Trimmel et al., 2018). Foscolo and colleagues have studied in TLE patients, the anatomical structure of the uncinate and inferior longitudinal tracts given their important role in language and memory and the anatomical proximity to the epileptogenic zone of patients. Consistent with the “minimal common brain” hypothesis (i.e., a fixed macrostructural connectivity, even in the case of severe brain damage; Ius et al., 2011), no anatomical differences were found between TLE patients and controls regarding the length or direction of these tracts. However, the WM microstructural modifications were detectable (Foscolo et al., 2007). Diffusion tensor imaging (DTI) studies performed in TLE patients indicate a global impairment of the WM microstructure, which may be driven by a progressive degenerative process in response to chronic seizures (Chiang et al., 2016, Keller et al., 2012). Although epilepsy is classically considered a cortical disease, DTI studies showed that WM fascicles are also affected (van Eijsden et al., 2011), even at a distance from the primary epileptogenic region as in the contralateral non-epileptic hemisphere (Arfanakis et al., 2002, Concha et al., 2005). DTI abnormalities are not confined to the epileptic temporal lobe but rather involve a large network of association, projection, and commissural WM fibers (Hatton et al., 2020).

The methodological progress in terms of acquisition and processing of DTI-MRI data is also substantial and allows to refine the past findings. The transition from relatively simple processing models such as diffusion tensor imaging (DTI) to more complex models such as Constrained Spherical Deconvolution (CSD) has led to considerable advances, particularly concerning the problem of fiber crossings (Tournier et al., 2007). The study of the topology, as well as the neurobiological nature of white matter damage in the pathological context (i.e., neurological disorders), directly benefits from these new approaches and techniques. Using the DTI model, Fractional Anisotropy (FA) reflects the WM microstructure, and its decrease indicates WM damage (Aung et al., 2013, Zhang et al., 2012). However, when WM fibers are not aligned (i.e., crossing fibers, kissing fibers) the DTI model is not appropriate to make interpretations (Jones et al., 2013). Yet, the fiber crossing problems may concern up to 90 % of brain voxels (Jeurissen et al., 2013). Overall, DTI has many advantages but is a too simple model to account for the neurobiological nature underlying WM changes. For example, patients with TLE exhibit aberrant neurogenesis in the vicinity of the seizure-generating region, resulting in atypical neoconnections or fiber sprouting (Ben-Ari et al., 2008, Cho et al., 2015). Beyond epileptic regions other structural changes are also frequently observed, such as the so-called “blurring phenomenon” (i.e., abnormalities in the boundary between gray and white matter; Naves et al., 2015). In these situations, interpreting WM abnormalities based on FA calculated with DTI models may be erroneous and underlines once again, the necessity of using more complex models to study WM microstructural changes induced by epilepsy.

In this context, the main objective of this study was to accurately characterize the WM damages in TLE patients. We used the CSD model (Tournier et al., 2007) to minimize the limitation related to the fiber crossings problem. By employing state-of-the-art deep learning tools, we performed probabilistic tractography of each individual and we extracted several quantitative microstructural parameters. To identify WM changes in patients, we applied a combined “big-picture and fine-grain” funnel approach. We performed several methods of dimension reduction, using both conventional and (non-linear) manifold learning statistical analyses. We first describe the global (i.e., the entire structural connectome) and tract-specific profile of impairments in patients, using clustering analyses to extrapolate information based on average patterns (profiling). We then refine and complete our profiling analyses by modeling and quantifying the inter-group/inter-individual variability in a reduced and relevant manifold space with TractLearn, a statistical pipeline optimized for these purposes (Attyé et al., 2021). Manifold techniques allow us to map the high-dimensional image domain to the reduced latent space of brain fascicles, and to handle high-dimensional low-sample size data (HDLSS; Hall et al., 2005). We also leveraged the manifold learning pipeline in terms of local anomaly predictions to target structural damage in patients at a finer scale (voxel-level).

As a second objective, we evaluated the effect of clinical, functional, and cognitive factors on WM reorganization. Current evidence converges on an association between the microstructural properties of white matter and cognitive deficits observed in patients with TLE (e.g., Leyden et al., 2015, Rodríguez-Cruces et al., 2020, Roger et al., 2018, for a review). However, the link between structural anomalies of WM and cognitive efficiency remains to be clarified given the high level of variability in TLE patients. Indeed, various degrees of cognitive deficits can be observed in these patients (Alessio et al., 2013, Hoppe et al., 2007, Jaimes-Bautista et al., 2015, Metternich et al., 2014), and functional reorganization patterns can be more or less cognitively efficient (Roger, Pichat, Torlay, et al., 2019). To estimate in depth the links between quantitative structural parameters, function, cognition, and clinical variables, we performed several correlational analyses on global and local estimates of structural changes identified in patients.

## 2 Material and methods

### 2.1 Populations

MRI scans of 40 healthy controls (HC) and 25 matched drug-resistant epileptic patients presenting unilateral TLE have been initially examined. We excluded subjects with movement artifacts making it impossible for DWI postprocessing (n = 3 HC; n = 7 TLE). In all, 37 HC (23 males; age 38.3 ± 7.1 years) and 18 TLE patients (10 with left TLE [LTLE]: age 34.95 ± 9.6 years; 8 with right TLE [RTLE]: age 36.39 ± 9.4 years) were finally considered. Patients, as well as controls, have provided written informed consent for the study that was approved by the local ethic committee (CPP: 09‐CHUG‐14, 04/06/2009).

Diagnosis of drug-resistant TLE was established by neurologists working in a specialized epilepsy care unit. This diagnosis follows the recommendations of the International League Against Epilepsy (ILAE) committee report (Wieser, 2004) and is based on the synthesis of several evaluations (clinical, intracranial EEG, MRI/PET scan). Patients enrolled in the study did not present other neurological comorbidities (traumatic brain injury, stroke, or tumors). One-third of them (35.6 %) had a febrile seizure history. They were candidates for future neurosurgery (temporal lobe resection) and have never had neurosurgery in the past, MRI evaluations were thus performed at the presurgical stage.

### 2.2 Clinical and cognitive description

**Fig. 1:**
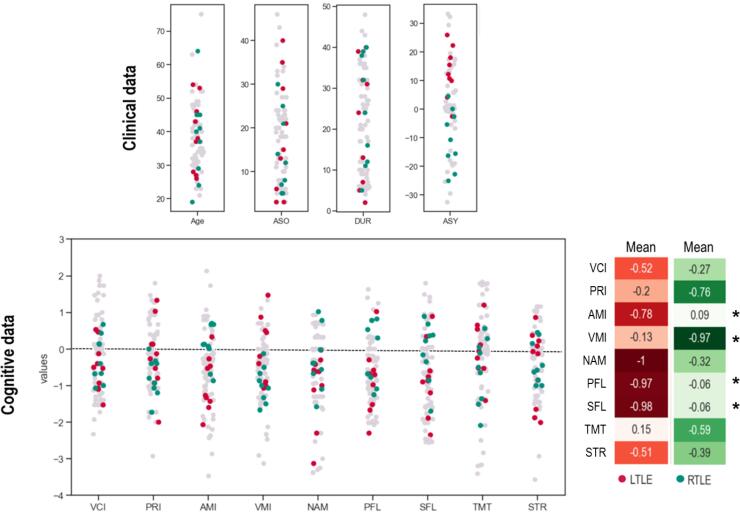
**Clinical and cognitive features of TLE patients.** Distribution of the LTLE (in red) and RTLE (in green) patients across several clinical (upper panel) and cognitive (lower panel) variables. For descriptive and comparative purposes, data from a previously published article aiming to identify cognitive fingerprint on a larger sample of TLE patients (left and right; n = 57; Roger et al., 2020) are projected in background (grey dots). Overall, patients included in this study present a similar profile to that of the larger TLE sample. LTLE/RTLE patients are matched on the clinical variables, also for the volume asymmetry between hippocampi (ASY; considering the absolute value). We observed significant differences at p <.05 on some cognitive variables (marked with a star, namely: AMI, VMI memory scores and SFL and PFL verbal fluency scores). However, these differences did not persist at a corrected statistical threshold. See main text for description of variables and associated statistics.

Several demographic and clinical features were considered such as: age (AGE); gender (GEN); educational level (EDU); age of epilepsy onset (ASO); duration of epilepsy (DUR); seizure frequency (FRQ); number of daily-taken antiepileptic drugs (AED); hippocampal sclerosis reported in clinical neuroradiology reports (HS); and asymmetry between hippocampi (ASY; estimated by the Volbrain protocol: https://volbrain.upv.es/; (Manjón & Coupé, 2016). On average, patients with LTLE and RTLE were matched regarding their clinical features: AGE (35,67 ± 9.5 years​; U = 43.5, *p* =.5); ASO (14.7 ​± ​10.5; U = 37.5, *p* =.3); DUR (12.2 ​± ​10.3; U = 36.5, *p* =.3); FRQ (U = 37.5, *p* =.3) and AED (U = 36, *p* =.2). TLEs were in addition comparable regarding their absolute value of ASY (U = 45, *p* =.5; Fig. 1).

We also included a hemispheric lateralization index (LI) derived from task-fMRI as these participants also underwent fMRI evaluation of language and memory networks. The LI is typically included in the clinical workup of drug-resistant epileptic patients at our Center. In practice, LIs were extracted from the functional activations during the sentence generation task with implicit encoding (language-memory task) of the GE2REC fMRI protocol (Banjac et al., 2020). They were measured based on frontal activation, owing to the very high level of congruence with the Wada lateralization test for language (Lehéricy et al., 2000). Operationally, we have used a mask based on symmetrical left–right anatomical frontal regions (Tzourio-Mazoyer et al., 2002, Tzourio-Mazoyer et al., 2017). We calculated frontal LIs for each patient and the control group, according to the method proposed by Seghier (2008). We used absolute LI values in this study, reflecting either no frontal specialization (LI close to 0) or left or right hemispheric specialization (LI above 0.4 and close to 1).

In addition, all patients have undergone complete cognitive assessment including neuropsychological and language testing, carried out by neuropsychologists and speech therapists from the neurology department. The following cognitive features were used as cognitive scores of interest: (a) general assessment: verbal comprehension index (VCI) and perceptual reasoning index (PRI; WAIS-IV, Wechsler, 2011); (b) language scores composed of naming (NAM; Deloche et al., 1997) as well as phonemic (PFL) and semantic (SFL) verbal fluency (Godefroy, 2008); and (C) memory scores composed of auditory memory index (AMI) and visual memory index (VMI) (WMS IV; Wechsler, 2012); (c) executive scores: Stroop test (STR) and trail making test (TMT; (Godefroy, 2008)). All raw scores were standardized into z scores by neuropsychologists; according to gender, age, and/or sociocultural level, as specified in respective manuals. We observed significant group differences at *p* <.05 by using parametric testing (two-sample t-tests) for AMI (*t*(17) = -2.49, *p* =.02), VMI (*t*(17) = 2.56, *p* =.02), PFL (*t*(17) = -2.16, *p* =.05) & SFL (*t*(17) = 2.12, *p* =.05) respectively. However, these differences did not survive at either a corrected threshold for multiple comparisons or by using Mann-Whitney non-parametric tests. Distributions of the standardized clinical and cognitive scores are presented in Fig. 1 below. Detailed descriptions of variables and statistical tables are included in supplementary materials (Appendix S1).

## 3 Diffusion MRI

### 3.1 Diffusion MRI acquisition

MRI was performed on a Philips Achieva 3.0 T dStream (Philips Healthcare**®**, Best, The Netherlands) with a 32-channel head coil. For the diffusion-weighted acquisition, we used a b-value of 1000 s/mm^2^, 60 diffusion-weighting non-collinear directions, and one b = 0 acquisition. Other parameters of the diffusion sequence were: TE/TR = 70 ms /11374 ms; Scan Time = 907 s (11′56″). To minimize artifacts, two additional b = 0 s/mm2 images were acquired before each HARDI acquisition. Each of the additional b = 0 s/mm2 images had identical imaging parameters as above, but one had its phase encoding reversed to allow susceptibility distortion correction (Holland et al., 2010). T1-weighted anatomical images were acquired using a 3D T1-TFE sequence with the following parameters: 256 × 256 × 160 matrix; 1 mm isotropic resolution; 256 × 256 within slice imaging matrix, TE/TR = 4.6 ms / 9.552 ms; flip angle 8°.

### 3.2 Diffusion MRI processing

• *Data pre-processing and tracts segmentation*

Preprocessing of diffusion-weighted images included denoising of data (Veraart et al., 2016); eddy current correction and motion correction (Andersson et al., 2003) as implemented in FSL (topup command; Smith et al., 2004); bias field and Gibbs artifacts’ corrections (Tustison et al., 2010), and up-sampling DWI spatial resolution by a factor in all three dimensions using cubic b-spline interpolation, to a voxel size of 1 mm^3^ (Raffelt et al., 2012a).

From the preprocessed data, we have estimated fiber orientation distributions using the Constrained Spherical Deconvolution model (CSD; Tournier et al., 2007) with the individual response function (RF) and by using multi-tissue (3-tissue) CSD variants (Dhollander et al., 2019). We derived the SH peaks from the fiber orientation distribution function (FOD) maps. All preprocessing steps were conducted using MRtrix3 commands (https://www.mrtrix.org) or using MRtrix3 scripts that interfaced with external software packages. For an efficient, automatic, and fast tracts segmentation, we used TractSeg (Wasserthal et al., 2018; openly available at: https://github.com/MIC-DKFZ/TractSeg). TractSeg algorithms propose to create bundle-specific tractograms by tracking directly on the tract orientation maps (probabilistic tracking) to perform tractography from original DWI data. Briefly, the authors have used convolutional neural networks for the tract segmentation, start/end region segmentation, and tract orientation mapping, all based on U-Net (Ronneberger et al., 2015) that receives FOD peaks as input.

• *Quantitative parameters extraction*

Overall and using *TractSeg*, 71 tracts were successfully and automatically segmented in all subjects. Note that we considered the individual parts of the corpus callosum (CC) separately and not the CC as a whole because of observations from previous studies showing differential alterations in subparts in patients with epilepsy (e.g., Weber et al., 2007).

From the segmented and binarized tracts, we extracted four quantitative parameters *via* MRtrix. We extracted fractional anisotropy (FA) values from the individual FA maps. We have also computed the apparent fiber density (AFD) coefficients (Raffelt et al., 2012b) from the FODs-related maps and using the afdconnectivity command. In addition, we generated track-weighted imaging (TWI) maps, containing streamline-related information (Calamante, 2017, Pannek et al., 2011). This allowed us to (a) reduce errors related to coregistration between subjects and minimize highly variable and artifact-sensitive cortical endings by retaining only 80 % of the values (see Attyé et al., 2021); and (b) consider additional contrast parameters for quantitative analyses that were shown to be sensitive and clinically valid (Calamante, 2016, Willats et al., 2014). The first contrast was based on the FODs amplitude along the direction of the tract (TWI-FOD or TWI; Willats et al., 2014). The second was the track-weighted version of FA (TWI- FA; Calamante et al., 2015). All quantitative metrics (FA, AFD, TWI-FOD TWI-FA) were extracted for each voxel contained in the tractograms.

### 3.3 Statistical analyses

We applied a statistical funnel approach to identify significant structural changes in TLEs relative to HCs from a global to local scale. We consider the tracts with respect to the hemisphere involved in the onset of epileptic seizures, reasoning in ipsi-epileptogenic tracts (ipsilateral to the problematic hemisphere) and contro-epileptogenic tracts (in the hemisphere contralateral to the epileptogenic focus identified by intracranial electrodes). For left TLE patients (LTLE), left ipsi-epileptogenic tracts were compared to their left counterparts in HCs and *vice versa* for right TLE (RTLE).

### 3.4 Estimation of global profiles

We averaged the voxel-wise quantitative values of each metric (independently) and for every tract. In all, we thus obtained 71 tracts mean values for all subjects (HC and TLE) and metrics (FA, AFD, TWI, TWI-FA, respectively). We first applied bootstrapped two-sample t-tests between groups on each bundle to identify robust and significant modifications in TLE. The statistical threshold of *p* <.05 was corrected for multiple comparison issues (Bonferroni correction). Significantly affected tracts in TLE compared to HC (i.e., tracts with a significant reduction/increase) were used as *fascicles of interest* (FOIs) for subsequent analyses.

To identify clusters of tracts presenting similar WM changes in TLE (global patterns of change), we performed hierarchical clustering on the FOIs. The distance matrix was built from centered values (TLE-HC) and was based on Euclidean distance. Convergence of clusters across measures was also tested by applying hierarchical clustering on the correlation matrices (Appendix S2, Figure S3). In addition, we carried out confirmatory analyses by using scikit (https://www.scikit-yb.org/en/latest/api/cluster/index.html) to (i) validate the optimal number of clusters with the elbow method; and (ii) assess the consistency and robustness of identified clusters using silhouette and inter-cluster distance analyses respectively (Appendix S2, Figure S2).

### 3.5 Statistical learning to assess variability

We applied a previously published manifold learning pipeline (TractLearn: Attyé et al., 2021; https://github.com/GeodAIsics/TractLearn-WholeBrain). TractLearn used geodesic manifold learning as a data-driven learning task and allows a unified framework for brain fascicles quantitative analyses. As TractLearn is integrated into a Riemannian atlas framework, f(x) represents the local mean value (i.e., the mean value of the closest HC) in the manifold space (Lawrence, 2003, Titsias and Lawrence, 2004). This way, the TractLearn algorithm has strong advantages in the detection of both global and local variability (Attyé et al., 2021, for a demonstration and a comparison with classical GLM to detect subtle differences).

As a first step of the pipeline, we reduced the dimensionality of all voxels contained in each tract to obtain one point per tract and per individual in the manifold space. *TractLearn* uses U-map (McInnes et al., 2018) as a dimension-reduction technique to preserve as much as possible the original structure of the data, in the reduced space (the points themselves and the euclidean/geodesic interpoint distances as well). An optimization-based method was applied to assess the optimal number of latent dimensions. Note that for illustrative purposes, however, we used a systematic 2D representation.

As a second step, we learn the manifold based on the HC data [Y = f(x) + ε]; where Y represents the patient in the “original” tract space (i.e., quantitative values extracted from each TractSeg tract), x the corresponding point in the HC reduced space, ε are the residuals and represent the subjects’ variability. f is thus the regression equation between the reduced space and the real space. TLE patients were individually projected into the reduced space of HC (x) and then back-projected (f--1 kernel regression) into the tract space (f(x)).

Placed in the reduced manifold space, we have then computed the Kullback-Leibler divergence (DKL) measure between TLE and HC to assess statistical divergence (inter-group/ inter-individual variability). We used a bootstrap procedure and applied a Bonferroni-like correction to adjust the statistical threshold of significance for multiple comparison problems (*p* <.001). The pipeline also allows to posit one single patient relatively to others (HC and/or TLE) and to assess the inter-/intra-individual variations of individual TLE patients, compared to HC.

### 3.6 Statistical learning to predict local anomalies

In addition to detecting inter-individual divergences, we used TractLearn to finely target WM abnormalities in TLE. We calculated residuals ε in the subject space to identify voxels with significant modifications compared to the HC norm, employing z scores. ε was estimated for each patient using a leave-one-out strategy. Voxels with a z score greater/lower than a Bonferroni adjusted threshold (≅ ∓ 4.8 SD) were considered significantly deviant.

We used the thresholded z scores maps of alterations as an input to measure a relative percentage of “lesion” (%RL = number of deviant voxels / total number of voxels contained in the tract) for each tract and patient. We merged the respective tract-based maps to obtain whole-brain lesion maps for each individual. Individual maps were also subsequently aggregated to map the probability of WM lesions at the group level.

### 3.7 Connecting structural metrics to clinical and cognitive features

To identify the association between clinical/cognitive features to FOIs parameters at the global scale (connectome and tracts), we performed classical correlation analyses (Spearman correlations) and we used a conventional statistical threshold of significance of p <.05. We further estimate the associations between the global structural profile and clinical/cognitive factors by taking advantage of the manifold. Manifold analyses are particularly useful for observing and taking variability into account. Here, we have sought to make sense of the latent dimensions by using a similar approach to Renard et al., (2021). More specifically, logistic regression was applied in the reduced space produced by U-map to color-code the probability of belonging to a class of a given cognitive and/or clinical variable. The resulting projection provides insight into whether and how this variable describes the data in the reduced space.

Finally, we used NiiStat scripts (https://github.com/neurolabusc/NiiStat) to map the lesion-behavior correlations at a local scale (i.e., at the voxel level; VLBM). Bivariate testing was based on 5000 permutations. We set the minimum of overlap at 6 – meaning that we considered only damaged voxels present in at least one-third of patients – ensuring that correlation analyses are performed in regions typically impaired in TLE and avoiding contamination by low-power voxels (Sperber & Karnath, 2017). All statistics of the multimodal investigations were systematically adjusted for multiple comparisons.

## 4 Results

### 4.1 Structural modifications in patients

#### 4.1.1 General trends of structural damage

**Fig. 2:**
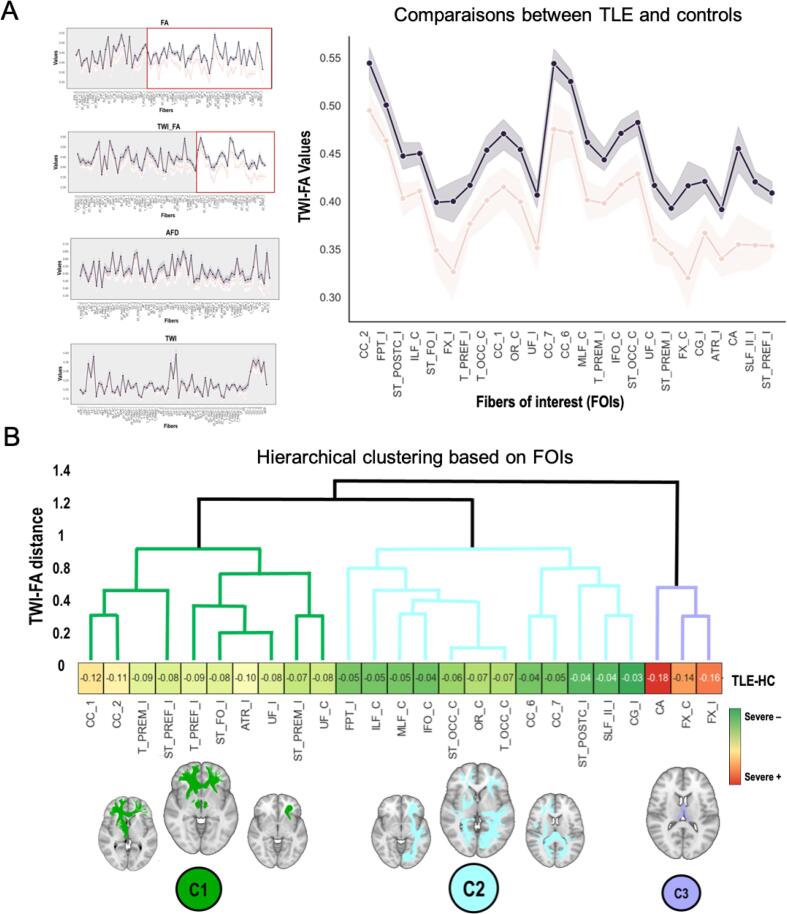
**WM microstructure changes and tract-based patterns of alteration A.** At left, the TLE-HC comparisons on the 71-tract mean values (independently) estimated from the following metrics of interest: AFD, FA, TWI, TWI-FA. Significant differences between TLE-HC at p <.05 (with Bonferroni correction ≅ p <.001) are framed in red (See Appendix S2, Figure S1 for enlarged version of the plots.). We found FA and TWI-FA modifications in TLE compared to HC. TWI-FA identify more specifically the alterations than the traditional FA metric. At right, the line plot of the mean TWI-FA of tracts presenting significant differences between TLE and HC (fascicles of interest, FOIs). **B.** Hierarchical clustering of the FOIs based on the distance matrix of the centered TWI-FA values (dendrogram of Euclidean distance). 3 main clusters of damage were identified at the second level of the dendrogram (C1: ipsilateral fronto-basal fascicles; *C*2: contralateral fascicles with posterior projections; C3: bilateral temporo-mesial tracts).

We found significant differences between TLE and HC for two of our four quantitative metrics: the FA (44 tracts) and the TWI-FA (25 tracts). The TWI-FA measure being sensitive and more specific (Fig. 2), we focused the rest of our analyses on this metric.

TLEs present a systematic decrease in TWI-FA when compared to HCs (no increase; see Fig. 2, panel B). Tracts presenting a significant reduction in TWI-FA (p <.05 Bonferroni corrected) in TLE versus HC are the: uncinate fascicle (UF), cingulum (CG), superior longitudinal fascicle branch II (SLF_II), fronto-pontine tract (FPT), fornix (FX), anterior thalamic radiation (ATR), thalamo-prefrontal (T_PREF), striato-prefrontal (ST_PREF), thalamo-premotor (T_PREM), striato-premotor (ST_PREM), striato-postcentral (ST_POST), striato-fronto-orbital (ST_FO), for ipsi-epileptogenic tracts; the uncinate fascicle (UF), inferior longitudinal fascicle (ILF), inferior occipito-frontal fascicle (IFO), fornix (FX), middle longitudinal fascicle (MLF), striato-occipital (ST_OCC), thalamo-occipital (T_OCC), optic radiation (OR), for contralateral tracts; and the rostrum, genu, isthmus, splenium of the corpus callosum and the commissure anterior (CC1, CC2, CC6, CC7, CA respectively), for interhemispheric fibers. These 25 tracts are considered fascicles of interest (FOIs) in subsequent analyses (Fig. 2).

**Fig. 3:**
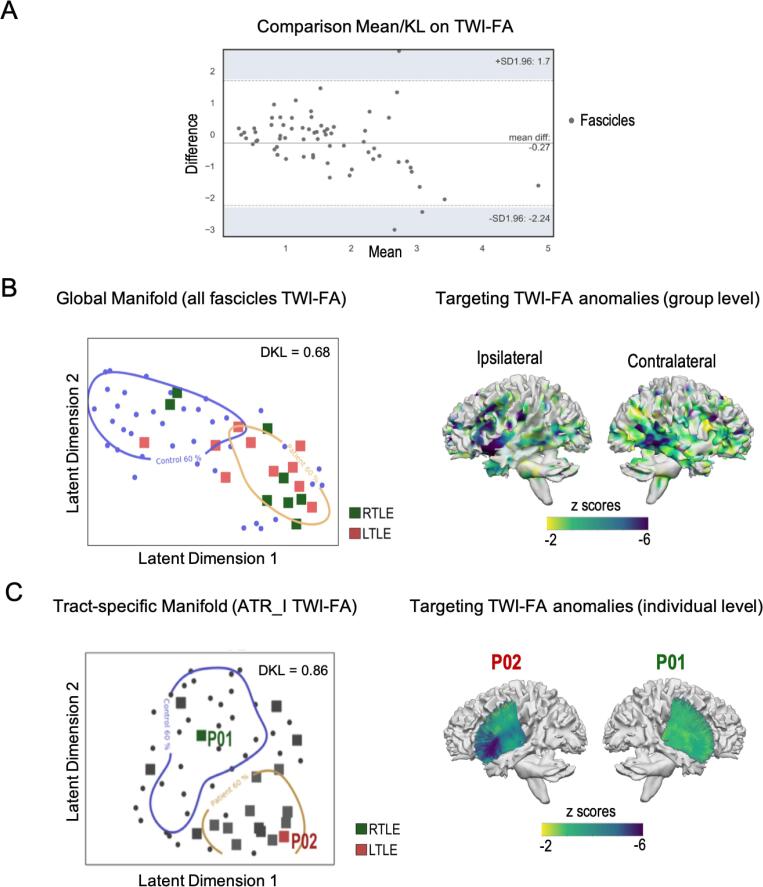
**Manifold and voxel-wise approaches at the interface between group differences and individual alterations in patients.A.** Altman plot, quantifying the agreement between two measures. The closer the difference is to zero, the better the agreement. The average difference between the *t*-values obtained from traditional statistics and the KL divergence values extracted from the manifold space is −0.27 for TWI-FA. Note also that the average differences regarding the other parameters are: FA = 0.36; AFD = 0.24; TWI = -0.29.**B.** At left, the global 2D manifold based on the 25 FOIs (HCs in blue; RTLE in green; LTLE in red). The distance between controls and patients is significant at p <.05. At right, the map of lesions observed at the voxel level in the TLE group, using TractLearn. The map is projected on a WM template. Voxels with a significant reduction in TWI-FA after Bonferroni correction (z < -4.8) are displayed in purple.**C.** At left, the 2D manifold of a given FOI (Anterior Thalamic Radiation ipsi-epileptogenic: ATR_I) as an example, showing the relative position of individual patients (P01, P02) compared to the control group (in blue). At right, the lesion maps of these two patients projected on the template of the corresponding fascicle, highlighting both inter- and intra-individual variability.

The hierarchical clustering performed on the FOIs and based on the similarity of TWI-FA changes converge onto 3 main patterns of alteration in patients (Fig. 3, Panel B). Cluster reliability analyses (inter-cluster distance and internal consistency) were performed and are presented in Appendix S2 (Figure S2). Overall, the 3-cluster solution categorizes tracts according to the following scheme: fronto-basal fascicles (C1, mainly ipsilateral), bundles with posterior projections (C2, mainly contralateral), and temporo-mesial tracts (C3, bilateral); with C3 > C1 > C2, from the most to the least severe alterations in TLE (Fig. 2). Hierarchical clustering based on correlation matrices yielded similar results (Appendix S2, Figure S3).

#### 4.1.2 Interindividual variability

We found a good agreement between the mean differences of TWI-FA extracted from traditional analyses and the KL mean distances deriving from manifold analyses, with a global difference close to zero (d = -0.27; Fig. 3, Panel A). Moreover, by using the KL divergence (p less than 0.05 adjusted), we observe the same inter-group differences as those found with conventional tests (i.e., the 25 FOIs for the TWI-FA). It supports that the KL distance estimated in the reduced manifold space is a suitable measure to estimate inter-group or individual differences.

Manifold analyses complement conventional statistics. We found substantial changes in TWI-FA in TLEs compared to HCs, involving more than twenty WM tracts. The inter-group divergence, estimated in the manifold that includes the 25 FOIs (global manifold) is high (DKL = 0.68; Fig. 3 Panel B) but no longer significant after correction for multiple comparisons (p <.001). There is indeed some interindividual variability that is finely captured by the manifold. In the reduced manifold space, some TLE patients deviate from the norm while others show an overall TWI-FA pattern similar to that of HCs, which limits conclusions at the group level. Even on a single FOI, i.e., on a specific tract detected as significantly affected, inter-individual divergences can be notable. Fig. 3 Panel C shows the example of one FOI – the ipsilateral anterior thalamic radiation (ATR_I) belonging to C1 (Fig. 2) – that demonstrates a robust and significant inter-group divergence (mean DKL = 0.86, p <.001). Again, we observe some quantifiable interindividual variability in the ATR_I manifold space. For example, the RTLE patient P01 has a mean z-score of anomalies of −0.07 and is globally among controls, whereas P02 (a patient with LTLE) deviates significantly from the norm with a mean z-score of −2.56 for the same tract.

#### 4.1.3 Local structural anomalies

Manifold analyses can also be used to target local structural anomalies. The TWI-FA lesion map for the TLE group is projected in Fig. 3 Panel B and shows the localization of anomalies calculated at the voxel level (per voxel z scores). For each FOI, the average relative percentage of lesions (TWI-FA) varies around 5 % of the total number of voxels contained in the given tract (statistical threshold z < -4.8). It involves contiguous voxels and generally less than 10 % of the FOIs (Appendix S2, Figure S4). Thus, it is more a certain part of the tract than the entire tract that is significantly affected. In addition, lesion z-score maps can be estimated at an individual level. Fig. 3 Panel C shows individual TWI-FA lesion maps (z-scores voxels for P01 and P02), projected onto the ATR_I tract for illustrative purposes.

### 4.2 Link with cognitive and clinical data

**Fig. 4:**
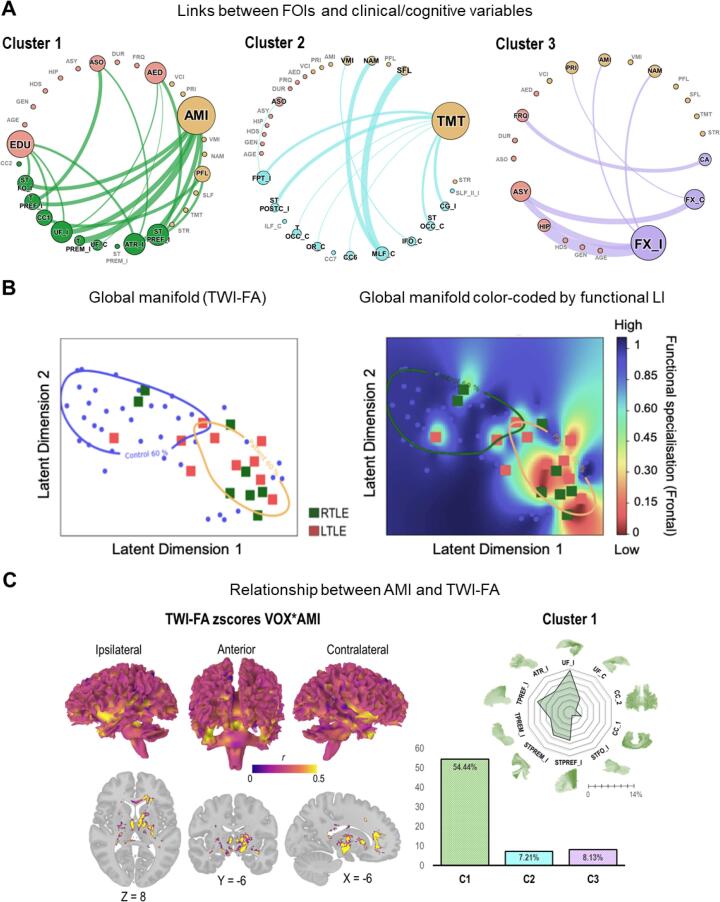
**Relationships between structural changes and clinical, functional and cognitive variables, at different levels A.** Relational networks between clinical/cognitive data and tracts according to the main clusters (circular layout). The illustration shows thresholded correlations at p <.05. Only the connections between FOIs and clinical or cognitive variables are presented. Inter-connections between clinical and cognitive categories or within categories are not represented. The size of the nodes corresponds to the degree of interconnectivity (i.e. the number of connections between the node and the other nodes of the network). The larger the node, the more the variable is connected to other variables. Nodes are color-coded as follows: cognitive variables are in orange; clinical in red; and FOIs in their respective cluster colors (C1 = green, C2 = blue; C3 = purple; Fig. 2B). Labels for disconnected nodes (no significant connection in the network, degree = 0) are written in gray. Table S2 (Appendix S2) reports the correlation values (p <.05).**B**. Color-coded probability map of the functional lateralization index (LI) projected into the global manifold (see also Fig. 2B). The color gradient from blue to red corresponds to the probability of exhibiting a highly lateralized pattern of activations during the sentence generation task (left and right hemisphere predominance; values near 1) versus a weakly lateralized or bilateral pattern (values near 0).**C.** At left: voxel-based lesion-behavior statistical mapping (VLBM; altered voxels * AMI correlations), projected on a 3D WM template and multiplanar slices. Voxels that are significantly correlated with the auditory-verbal memory (AMI) scores after permutations and corrections are shown in yellow (r > 0.5). At right: distribution of the correlated voxels across the 3 main clusters. The bar plot represents the relative proportion of impaired voxels (z TWI-FA < −4.8) correlating with AMI z scores, namely: C1 = 2581/4741 = 54.44 %; C2 = 410/5690 = 7.21 %; C3 = 66/811 = 8.13 %. The spider plot represents the distribution of altered voxels correlated to AMI (relative proportion), with a focus on the FOIs of the cluster 1 that is strongly related to AMI (see bar plot and Fig. 4 Panel A).

We found significant correlations between some clinical and cognitive variables and TWI-FA values of specific FOIs belonging to the cluster C1, C2 or C3. For the C1 FOIs (ipsilateral fronto-basal fascicles), AMI (r = 0.8) and PFL (r = 0.77) for cognitive indicators; EDU (r = 0.62), ASO (r = 0.66) and AED (r = 0.55) for clinical factors were significantly correlated (p <.05). Only some cognitive variables were significantly correlated with the C2 FOIs (contralateral tracts with posterior projections), namely: SLF (r = 0.54), NAM (r = 0.48) and TMT (r = 0.63). Finally, we found significant relations between bilateral temporo-mesial tracts (C3 FOIs) and AMI (r = 0.46) or NAM (r = 0.46) cognitive scores; and ASY (r = 0.67) or HIP (r = 0.56) clinical attributes. Fig. 4 Panel A shows the specific relational links (significant correlations, p <.05) between individual tracts belonging to the different FOIs clusters and cognitive/clinical features.

Complementing traditional analyses, we observed a strong association between the global manifold (Fig. 3) and the absolute lateralization index (LI) of the fMRI activity of the frontal lobe during the sentence generation task. Fig. 4 Panel B illustrates the probability color-coded interpolation map deriving from the reduce manifold space**.** The lower the functional LI (i.e., the frontal hemispheric specialization), the more spatially distant patients tend to be from the HC norm. In particular, the latent dimension 1 of the reduced space is well organized by the gradient of the functional LI. TLEs presenting the largest structural deviations also tend to show a more bilateral frontal functional profile on the language task. Tract-specific analyses have revealed that the TWI-FA values of the genu of the corpus callosum (CC2) were significantly correlated with frontal LI (at a threshold corrected for multiple comparisons; r = 0.78, p <.001). TWI-FA of the CC1 (the rostrum of the corpus callosum) and the ATR_I (the ipsilateral anterior thalamic radiation) also correlate with LI values but at an uncorrected p <.05 threshold (r = 0.59; r = 0.57, respectively). Thus, the anterior part of the CC may be a determining anatomical region in the structure–function relationship observed here.

Finally, we found an association between the voxels that significantly diverge in the global manifold space (Fig. 3 Panel B) and the AMI cognitive score (mean r = 0.61; Figure S7, Appendix S2). Fig. 4 Panel C illustrates the local spatial correlations or “lesion-behavior mapping” of affected voxel * AMI.

Significantly correlated voxels were extracted and labeled according to the cluster they belong to (C1, C2, or C3). In agreement with the correlation analyses performed at the tract level, C1 is overrepresented (in terms of the relative proportion of voxels) and some C1 tracts tend to be more involved than others (descriptively, UF_I, TPREF_I, ST_PREF_I, and STREM_I; Fig. 4 Panel C).

## 5 Discussion

In the present study, we aimed at assessing structural changes in WM tracts in a population of epileptic patients typically suffering from chronic damage to brain networks. Instead of using the classical DTI model for our tractographic analyses, we used an advanced CSD model to minimize the so-called “crossing fiber problem” (Schilling et al., 2022, Tournier, 2010). In line with previous reports, our results indicate a widespread decrease in FA in patients compared to controls, involving a vast majority of their WM tracts (Fig. 2). For example, Hatton et al., (2020) have also recently shown a significant and global FA decrease in the hundreds of TLE patients comprising the ENIGMA cohort. It involved multiple association and interhemispheric tracts in both hemispheres, corresponding to the cortical changes described in the same patients (typically within temporo-mesial, superior frontal, pre- and post-central gyri, as well as thalamic regions; Whelan et al., 2018). Meta or mega-analyses using DTI-FA thus conclude to a systemic impairment of the connectomic microstructure, even though the seizures are initiated by a (more or less) focal point in the temporal lobe.

However, more complex tractometric parameters considering fiber-related information (tract-weighted contrasts using for example the number of fibers contained in a voxel or the mean curvature; see Calamante, 2017), can be more sensitive and specific (see Fig. 2 Panel A). In this study, tract-weighted FA or TWI-FA has indeed shown a better specificity for the detection of WM abnormalities in patients compared to the traditional FA. Willats et al., (2014) demonstrated that TWI-FA provides a more realistic view of complex fiber architecture, where conventional FA measurement poorly reflects abnormalities within complex tracts. The clinical relevance of TWI-FA in reliably identifying severe (e.g., glioblastoma: Barajas et al., 2013) but also subtler brain damage (demyelination: Lyksborg et al., 2014) has also been demonstrated. Finally, we have previously observed a better agreement of test–retest measures at a one-year interval on the TWI-FA than on the FA in patients presenting mild traumatic brain injury (Attyé et al., 2021). Overall, several lines of evidence suggest that the TWI-FA contrast is useful for a finer and more reproducible detection of structural alterations in brain pathologies. However, the sensitivity of TWI-FA needs to be validated by specific studies. Indeed, as there is no gold standard to date, comparison of the predictive power of TWI-FA and FA to identify WM lesions on diverse populations is still needed.

Regarding the topology and the intensity of WM damage in TLE patients, we observed a gradient of structural dysfunctions with a more pronounced TWI-FA decrease near the epileptogenic networks, typically the WM of temporo-mesial and fronto-subcortical ipsilateral regions (Fig. 2 Panel B). This result is consistent with previous DTI findings that described a centrifugal decrease in impairment relative to epileptogenic networks (i.e., the spatial proximity; Concha et al., 2012), and in line with the “initiation” theory (Riley et al., 2010). Interestingly, Ellmore et al., (2011) showed that despite a significant reduction in the number of fibers connected to the epileptogenic hippocampus, TLE patients exhibit long-lasting functional hyper-connectivity between the hippocampus and the rest of the brain. The possible axonal loss in the temporo-mesial region remains to be further investigated with the recently developed tools and methodologies. Although the use of the CSD model minimizes the impact of fiber crossings in the tractogram study, the TWI-FA estimate does not allow a categorical conclusion about the neurobiological nature of the change (e.g., a decrease of TWI-FA may reflect demyelination but also axonal loss, or even mechanisms related to inflammation). It has been suggested, in animal models, that the ratio of axial to radial diffusion (AD/RD) might be sensitive enough to dissociate different types of damage (Zhang et al., 2012). Track-weighted versions of axial and radial diffusion maps could then provide an interesting future pathway to infer neurobiological mechanisms underlying WM changes using *in vivo* data. Preliminary analyses performed on our sample using AD and RD contrast maps (i.e., TWI-AD and TWI-RD) and focusing specifically on the temporo-mesial cluster of WM damage (cluster 3), might be in favor of “axonal degeneration” for proximal fibers (ipsilateral fornix and anterior commissure) and rather “demyelination” for contralateral fibers (contralateral fornix; see Table S3, Appendix S2 for statistical details). However, these first evidences remain to be confirmed by comparative studies jointly having anatomopathological data (i.e., surgical specimens from patients). Regardless of the nature of the damage, the “hippocampal paradox” (i.e., hyperfunctioning and/or hyper-connectivity despite damage; see Roger, Pichat, Torlay, et al., 2019), illustrates the great complexity of the structure–function relationship in epilepsy.

The second objective of our study was to investigate this relationship more globally, from the structure to the behavior, through the involvement of some clinical variables associated with epilepsy. Overall, TLEs with the greatest alterations in WM jointly show a decrease in functional frontal hemispheric specialization associated with language. Indeed, we observed a gradual transition between specialized (typical left hemisphere or atypical right hemisphere) and non-specialized (bilateral) functional patterns depending on the degree of WM integrity (Fig. 4B). By refining the analysis, we have observed that this relationship is notably driven by the integrity of the corpus callosum (Section 3.2). Specifically, the more the anterior part of the corpus callosum is affected, the more the functional specialization tends to be atypical and especially bilateral. These results are consistent with those reported in patients with corpus callosum agenesis (Hinkley et al., 2016) but they also suggest that a complete callosal disconnection is not necessary to induce functional specialization changes. Such a relationship between the integrity of the corpus callosum and changes in interhemispheric asymmetry was also reported for several cognitive processes (Schulte & Müller-Oehring, 2010) and, altogether, these observations support the idea that structural connectivity actively modulates functional wiring (Hervé et al., 2013).

Several clinical and cognitive factors are respectively related to the identified clusters of microstructural impairment (Fig. 4 Panel A). The severity of the WM damage of the temporo-mesial cluster (C3), and especially of the fornix (Fig. 4 Panel A), was associated with hippocampal volumes (HIP) and volume asymmetry between the two hippocampi (ASY; see Section 3.2). Abnormalities of WM microstructure are indeed more important in TLE than in patients with other forms of drug-resistant epilepsy, mainly when they suffer from hippocampal sclerosis (Hatton et al., 2020). Moreover, we found a strong relationship between these fibers and the associative and declarative long-term memory index of the WAIS-IV (AMI; Wechsler, 2012). The relationships between long-term (declarative) memory performance and mesial temporal structures have been pointed out for a long time in patients with severe hippocampal damage (see the pioneering work of Brenda Milner for example; Milner, 1966, Milner, 1970, Scoville and Milner, 1957), and the preservation of the MTL structural connectivity (the fornix in particular) plays indeed a crucial role in maintaining memory performances throughout life (e.g., Foster et al., 2019). WM changes of the fronto-subcortical cluster (C1) was related to the age of seizures onset (ASO), the number of antiepileptic drugs (AED), and the (phonological) verbal fluency (PFL). These are also classic observations in the epilepsy literature (Brissart & Maillard, 2018). The fact that these impairments were primarily related to the age of seizures onset but not to the duration of epilepsy suggests a developmental fragility of these structures (Lin et al., 2008) rather than a gradual pathological process related to the chronicity of epilepsy (Chiang et al., 2016, Keller et al., 2012), which should be further explored (Ashraf-Ganjouei et al., 2019). In addition, the relationship between frontal abnormalities, antiepileptic drugs, and verbal fluency may be intricate and it may be interesting to investigate whether medication mediates the relationship between cognition and the integrity of anterior structural connections. Finally, we found an interesting relationship between the contralateral posterior cluster (*C*2) and semantic cognitive indicators. Indeed, the middle longitudinal fasciculus (MLF) was related to naming (NAM) and semantic fluency (SFL) performance, according to recent findings showing similar correlations in patients with primary progressive aphasia (Luo et al., 2020) or stroke (Blom-Smink et al., 2020). This result suggests that the focus should be made not only on the anteroposterior association pathways, such as classically admitted (Bullock et al., 2019) but also on transverse dorsoventral pathways which are only more recently studied and that offer new perspectives to apprehend language-and-memory interactions (Hula et al., 2020).

From a methodological point of view, we used a funnel approach allowing us to investigate WM anomalies in a comprehensive and detailed manner. In this way, we observed that the bundles were not affected as a whole, nor homogeneously along the tract (Fig. 3 Panels B-C). Instead, we reported damage to segments or “portions” of tracts (Figure S5, Appendix S2). Some cognitive functions such as memory or functional networks such as the DMN (default mode network) do not engage entire anatomical tracts but rather specific fiber segments (Alves et al., 2019). Thus, accurate multimodal mapping between fibers and functional activity will provide greater insight into structure–function-cognition links (Nozais et al., 2021) and advance our understanding of neuroplasticity and its consequences in patients.

We leveraged the benefits of statistical manifold learning algorithms to finely profile, model, and predict WM abnormalities in patients. A major interest in using such a statistical learning model is to explore inter-/intra-individual heterogeneity (Fig. 3 Panel C; see also Figures S5-S6 in Appendix S2 for an example of interindividual variability in our sample regarding the distribution of abnormalities). Indeed, rather than assuming that all patients are reliably represented by measures of central tendency and *a priori*-defined groups, these tools can be used to directly capture and analyze patient heterogeneity (Cearns et al., 2019, Marquand et al., 2016). TractLearn, such as other software and method based on artificial intelligence (Chamberland et al., 2019, Chamberland et al., 2021), is a promising tool for moving toward precision medicine (i.e., more personalized and individual-oriented medicine). It has a strong potential of application and has also been shown to be robust in other populations, such as patients with traumatic brain injury (Attyé et al., 2021). More generally, machine learning techniques minimize priors and “human” influence in data management and analysis. On the other hand, the lack of an explicit model can make it difficult to directly link machine learning solutions to existing biological knowledge (Bzdok et al., 2018). Promising avenues in the interpretability of models, however, allow to make sense of black-box models and to move towards “white-box” models (Molnar, 2018). In our study, the addition of a clinical variable in the manifold space, for example, made sense of the observed latent dimensions (Fig. 4 Panel B).

## 6 Conclusions

Diffusion MRI provides parameters that are good predictors of postoperative outcomes in refractory epilepsy patients (Keller et al., 2017). In the presurgical stage, it can help to localize problematic networks since there is an apparent centrifugal decrease in damage relative to epileptogenic foci. Previous studies have indicated that structural WM MRI may also have a plus value and help to localize functional areas that should be preserved (e.g., De Witte et al., 2014, Roger et al., 2019a). Despite its utility, diffusion MRI remains rarely performed in clinical routines related to epilepsy care. From a practical standpoint and until recently, data analysis remained time-consuming and expensive. The development of advanced tools related to artificial intelligence now allows fast and efficient segmentation that facilitates data processing (e.g., TractSeg: Wasserthal et al., 2018) or automatic statistical analyses for anomaly detection (e.g., TractLearn: Attyé et al., 2021; Detect: Chamberland et al., 2021). Importantly, these tools – which are increasingly accurate, interpretable, and powerful compared to traditional models or human capabilities – are promising for bridging the gap between today's and tomorrow's personalized medicine.

## CRediT authorship contribution statement

**E. Roger:** Conceptualization, Data curation, Methodology, Formal analysis, Visualization, Validation, Writing – original draft, Writing – review & editing. **A. Attyé:** Methodology, Formal analysis, Software, Writing – original draft, Writing – review & editing. **F. Renard:** Methodology, Formal analysis, Software, Writing – original draft, Writing – review & editing. **M. Baciu:** Conceptualization, Writing – original draft, Writing – review & editing, Resources, Funding acquisition, Supervision, Investigation.

## Declaration of Competing Interest

The authors declare that they have no known competing financial interests or personal relationships that could have appeared to influence the work reported in this paper.

## Data Availability

Data will be made available on request.
