# Supplementary material for: Leveraging manifold learning techniques to explore white matter anomalies: An application of the TractLearn pipeline in epilepsy
Source: Neuroimage Clin. 2022 Sep 22;36:103209. doi: 10.1016/j.nicl.2022.103209 (PMC9668609; doi:10.1016/j.nicl.2022.103209)
Supplement: Supplementary data 1 [file mmc1.pdf]

**Appendix S1: Materials and method**

**Table S1: Clinical and cognitive information of TLE patients**

| ID | AGE | GEN | HDS | HEM | HS | HIP | ASY | ASO | DUR | FRQ | AED | EDU | VCI | PRI | AMI | VMI | NAM | PFL | SFL | TMT | STR |
|---|---|---|---|---|---|---|---|---|---|---|---|---|---|---|---|---|---|---|---|---|---|
| **Pat01** | 53 | Male | Right | Left | No | 4.49 | 4.1 | 22 | 31 | 1 | 2 | 2 | -0.4 | 1.33 | 1.87 | 1.47 | -0.62 | 1.02 | 0.89 | -0.53 | -0.85 |
| **Pat02** | 28 | Male | Right | Left | No | 5.1 | 0.4 | 21 | 7 | 1 | 1 | 2 | 0 | -0.13 | 1.33 | -1.13 | -0.62 | -2.3 | -2.5 | -0.25 | -2.01 |
| **Pat03** | 45 | Female | Right | Left | Yes | 1.96 | 25.1 | 5 | 40 | 3 | 4 | 2 | 0.67 | -0.33 | -0.87 | 0 | -0.39 | -0.68 | -1.7 | 0.56 | -1 |
| **Pat04** | 41 | Male | Right | Left | Yes | 3.11 | 10.76 | 30 | 11 | 2 | 2 | 2 | 0.14 | -0.4 | 0.12 | -1 | -0.02 | -0.61 | 0.06 | -0.5 | 0.45 |
| **Pat05** | 24 | Female | Right | Left | No | 4.6 | 0.02 | 12 | 12 | 1 | 2 | 1 | -0.68 | -1.2 | 0.01 | -1.5 | -0.56 | -1 | -0.12 | -0.65 | 0.01 |
| **Pat06** | 43 | Female | Right | Left | Yes | 2.77 | 10.67 | 3 | 40 | 3 | 3 | 3 | 0.53 | -0.27 | -0.27 | -0.2 | -0.42 | -2.01 | -2.35 | 0.65 | 0.08 |
| **Pat07** | 54 | Female | Left | Left | Yes | 2.04 | 25.87 | 15 | 39 | 3 | 2 | 1 | -0.93 | -0.53 | -0.2 | -1 | -0.03 | -0.7 | 1.2 | 0 | -0.13 |
| **Pat08** | 34 | Female | Right | Left | Yes | 2.88 | 17.96 | 29 | 5 | 3 | 2 | 2 | -1.07 | -0.8 | -1.07 | -1.2 | -6.43 | -0.67 | -0.6 | 0.54 | -1.88 |
| **Pat09** | 26 | Female | Right | Left | Yes | 2.5 | 15.42 | 13 | 13 | 3 | 3 | 3 | -0.53 | -2 | 0.27 | 0 | -2.3 | -1.52 | -1.89 | 1.79 | -1.65 |
| **Pat10** | 27 | Female | Right | Left | Yes | 3.13 | 12.14 | 3 | 24 | 2 | 2 | 2 | 0.46 | 1.03 | -1.43 | -0.4 | -1.12 | -0.98 | -0.9 | -1.41 | 0.22 |
| **Pat11** | 37 | Male | Right | Right | Yes | 2.77 | 16.31 | 21 | 16 | 2 | 2 | 2 | 0 | -1.73 | -0.67 | -0.67 | -1.1 | -0.53 | -0.38 | -1.36 | -0.58 |
| **Pat12** | 40 | Male | Right | Right | No | 5.09 | 5.42 | 8 | 32 | 1 | 4 | 3 | 0.13 | -0.93 | 0.07 | -0.33 | -0.58 | -1.25 | -0.16 | -1.51 | -1 |
| **Pat13** | 46 | Male | Right | Right | Yes | 2.04 | 22.2 | 40 | 6 | 3 | 4 | 2 | -0.13 | 0.13 | -0.53 | 0 | -1 | -0.98 | -0.87 | -0.06 | -0.07 |
| **Pat14** | 45 | Male | Right | Right | No | 4.12 | 4.45 | 7 | 38 | 1 | 3 | 2 | -0.67 | -0.8 | 0.67 | 0.13 | 0.67 | 0.78 | 0.89 | 0.28 | -0.45 |
| **Pat15** | 38 | Female | Right | Right | Yes | 3.96 | 9.87 | 6 | 32 | 2 | 2 | 2 | -1.53 | 0.13 | 0.47 | 0 | -0.58 | -0.58 | -0.76 | 0.11 | 0.86 |
| **Pat16** | 37 | Female | Right | Right | No | 4.01 | 2.61 | 35 | 2 | 1 | 1 | 2 | 0 | -0.93 | 1.33 | -2.07 | -0.3 | -0.3 | 0.34 | 1.2 | 0.37 |
| **Pat17** | 54 | Female | Left | Right | Yes | 3.70 | 15.6 | 25 | 29 | 3 | 1 | 2 | -0.4 | 0 | -0.69 | -0.87 | 1.02 | 0.82 | 0.68 | 0.05 | -0.15 |
| **Pat18** | 19 | Female | Left | Right | No | 4.24 | 2.67 | 14 | 5 | 2 | 3 | 2 | -1.27 | -0.4 | 0.7 | -1.07 | -0.39 | -1.09 | -0.86 | -0.11 | -0.85 |

## Clinical variables

AGE: Age of patient (in years)
GEN: Gender (Male/Female)
HDS: Handedness (Left/Right handed)
HEM: Hemispheric lateralization of the temporal lobe epilepsy (Left hemisphere; Right hemisphere)
HS: Hippocampal sclerosis (yes/no)
HIP: hippocampal volume in cm3 (ipsi-epileptogenic; whole structure), estimated using the VolBrain pipeline (https://volbrain.upv.es/instructions.php#volbrain_ppline)
ASY: Hippocampal asymmetry (absolute volume difference)
ASO: Age of seizures onset (in years)
DUR: Duration of the epilepsy (in years)
FRQ: Frequency of seizures (1 = a few seizures per month; 2 = one seizure per week; 3 = more than one seizure per week)
AED: number of antiepileptic drugs (daily taken)
EDU: Educational level (1 = < High School Diploma; 2 = < Bachelor; 3 = > Bachelor)

## Neuropsychological indices (NPE)

VCI: Verbal Comprehension Index (Wechsler, 2011)
PRI: Perceptual Reasoning Index (Wechsler, 2011)
AMI: Auditory Memory Index (Wechsler, 2012)
VMI: Visual Memory Index (Wechsler, 2012)
NAM: Naming DO80 (Metz-Lutz et al., 1991)
PFL: Phonological Fluency (Godefroy & GREFEX, 2008)
SFL: Semantic Fluency (Godefroy & GREFEX, 2008)
TMT: Trail Making Test (Godefroy & GREFEX, 2008)
STR: Stroop Test (Godefroy & GREFEX, 2008)

**Note.** The standardization of the scores obtained by patients (z scores) was carried out by the neuropsychologist with respects to the French norms, provided in respective manuals.
