# Supplementary material for: Leveraging manifold learning techniques to explore white matter anomalies: An application of the TractLearn pipeline in epilepsy
Source: Neuroimage Clin. 2022 Sep 22;36:103209. doi: 10.1016/j.nicl.2022.103209 (PMC9668609; doi:10.1016/j.nicl.2022.103209)
Supplement: Supplementary data 2 [file mmc2.pdf]

Appendix S2: Results

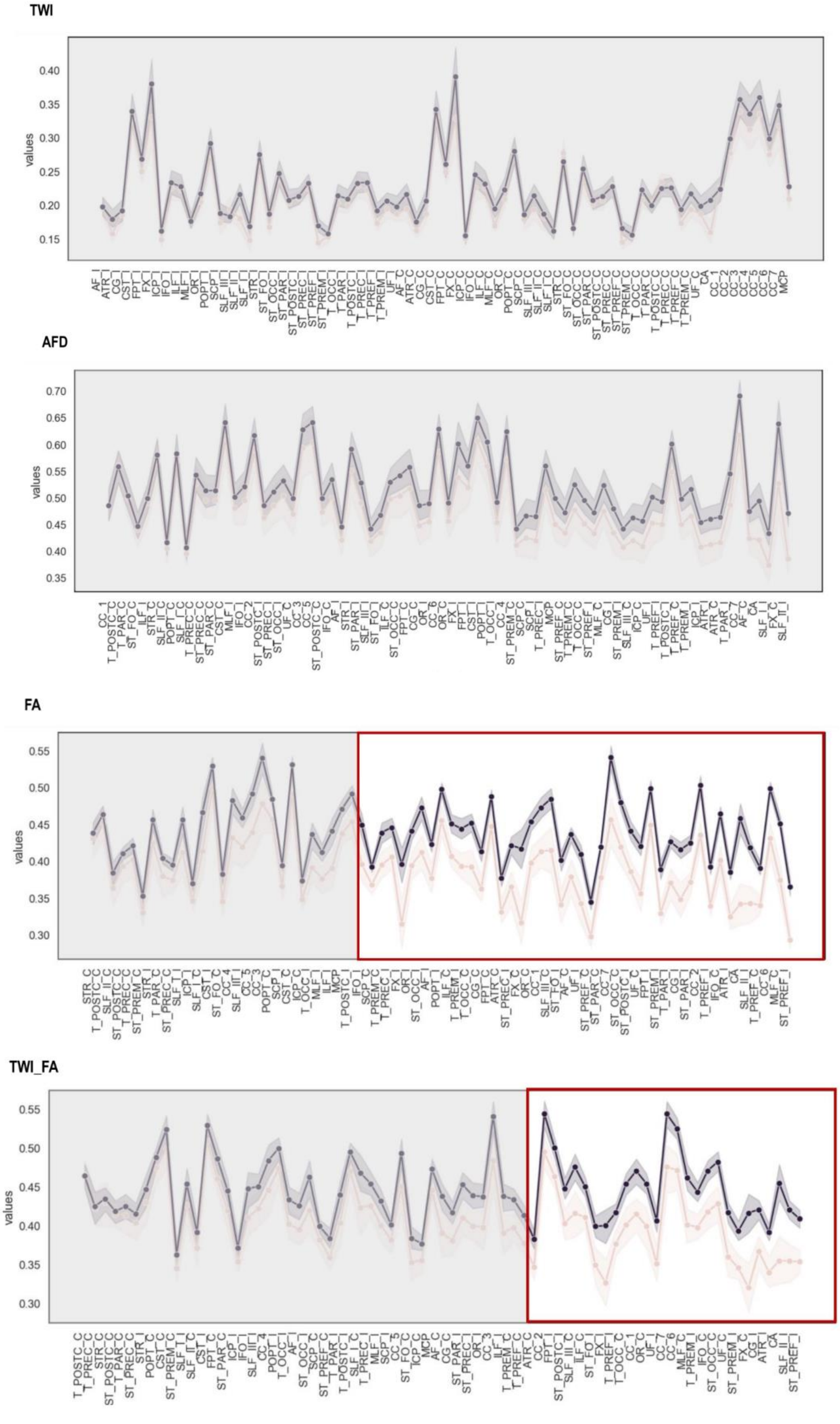

Figure S1: Statistical comparisons between TLE and HC on TWI, AFD, FA, TWI-FA

Significant differences between TLE-HC at $p < .05$ (with Bonferroni correction $\cong p < .001$) are framed in red

Table S1: Inter-measures correlations

Group All. Marked correlations are significant at p < .001 (N=55)

	Mean	SD	Mean TWI-FA	Mean TWI	Mean FA	Mean AFD
Mean_TWI-FA	0.43	0.05	1.00	0.41	0.83	0.40
Mean_TWI	0.22	0.05	0.41	1.00	0.38	0.78
MeanFA	0.42	0.05	0.83	0.38	1.00	0.40
MeanAFD	0.50	0.09	0.40	0.78	0.40	1.00

Group HC. Marked correlations are significant at p < .001 (N=37)

	Mean	SD	Mean TWI-FA	Mean TWI	Mean FA	Mean AFD
Mean_TWI-FA	0.44	0.05	1.00	0.36	0.88	0.29
Mean_TWI	0.23	0.05	0.36	1.00	0.31	0.83
MeanFA	0.43	0.04	0.88	0.31	1.00	0.26
MeanAFD	0.51	0.09	0.29	0.83	0.26	1.00

Group TLE. Marked correlations are significant at p < .001 (N=18)

	Mean	SD	Mean TWI-FA	Mean TWI	Mean FA	Mean AFD
Mean_TWI-FA	0.40	0.04	1.00	0.67	0.79	0.62
Mean_TWI	0.21	0.03	0.67	1.00	0.71	0.75
MeanFA	0.39	0.04	0.79	0.71	1.00	0.64
MeanAFD	0.48	0.08	0.62	0.75	0.64	1.00

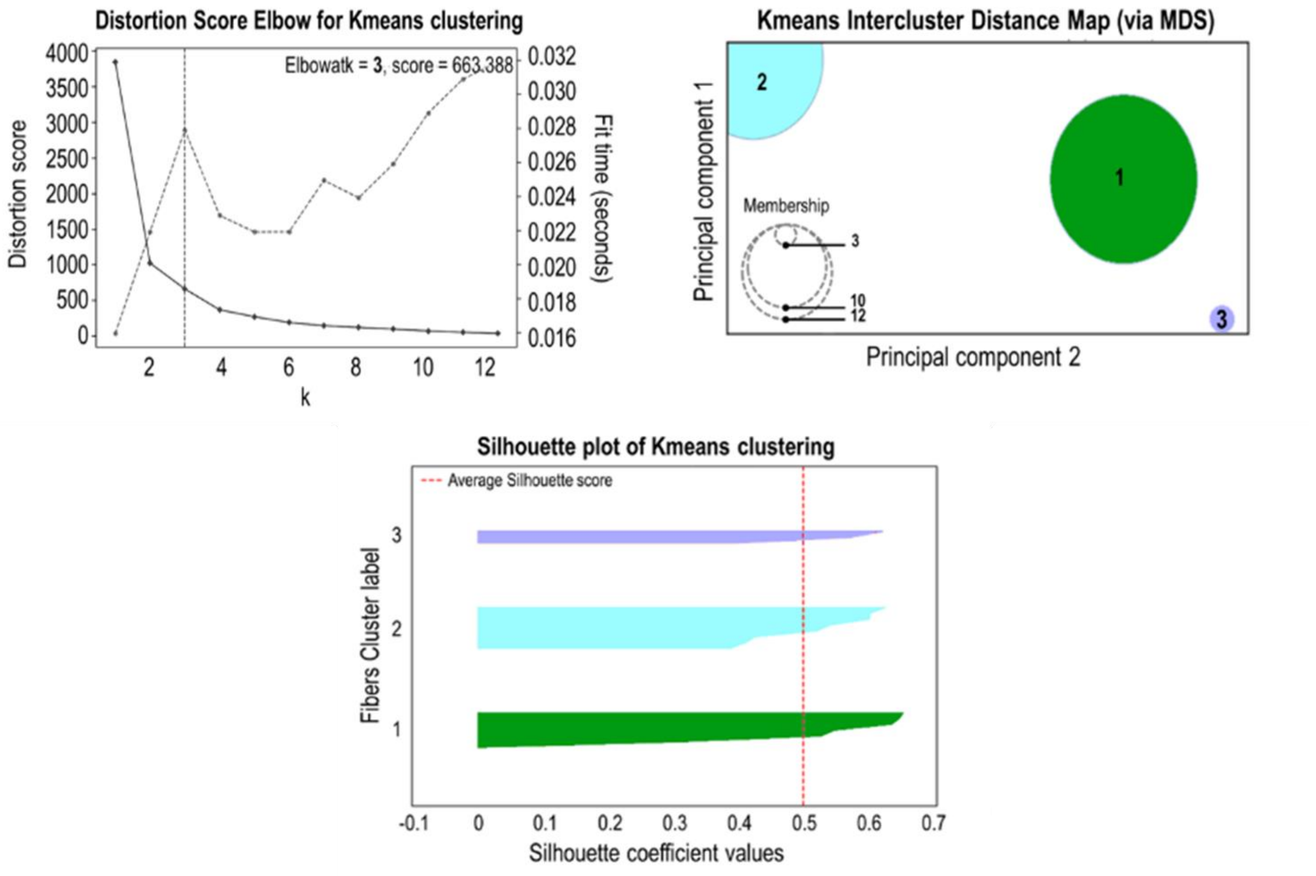

Figure S2: Confirmatory analyses of the stability and consistency of the identified clusters

The elbow plot confirms an optimal 3-cluster solution. The inter-cluster distance map illustrates the 2D-spatial segregation between C1, C2 and C3. The silhouette scores indicate the membership and intra-cluster stability of the FOIs into the 3 main clusters (silhouette scores could vary between -1 and 1, an average silhouette score of 0.5 indicates reliable internal consistency).

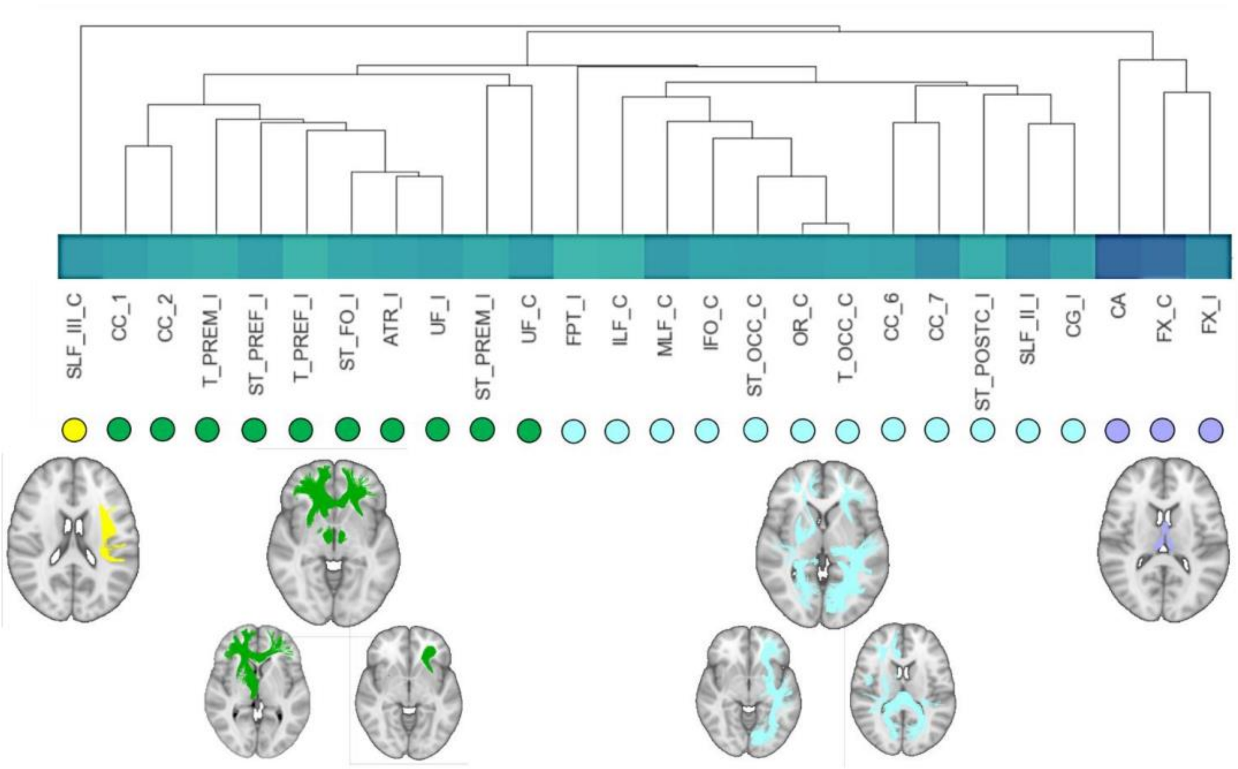

Figure S3: Hierarchical clustering applied on the correlation matrices

The dendrogram performed on the correlation matrices (Pearson corr.) shows some consistency with the clusters identified on the basis of the Euclidean distance (convergence between measures).

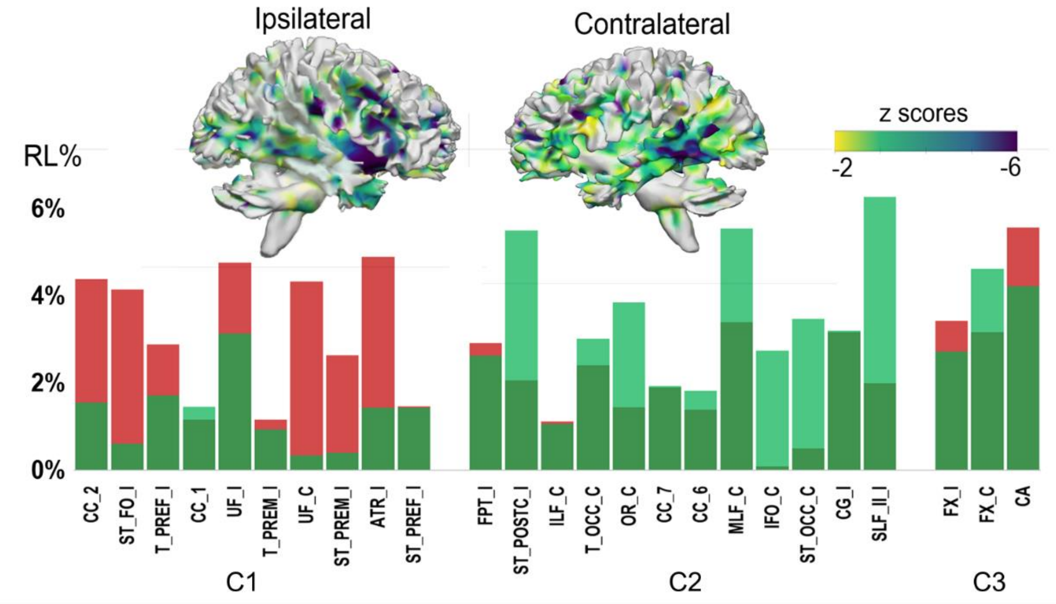

Figure S4: Percentage of alteration by tracts

Spatial detection of WM anomalies at the group level and by using TractLearn (Attyé et al, 2021). Spatial statistical maps of altered voxels in TLE versus HC (group level) are projected onto a template of WM surface. The relative percentage of altered voxels (%RL) in FOIs is based on TWI-FA (thresholded z scores). Descriptively, C1 appears to be more affected in LTLE (red) than in RTLE patients (green). Conversely, C2 seems more affected in RTLE than in LTLE patients. There is no clear trend for C3.

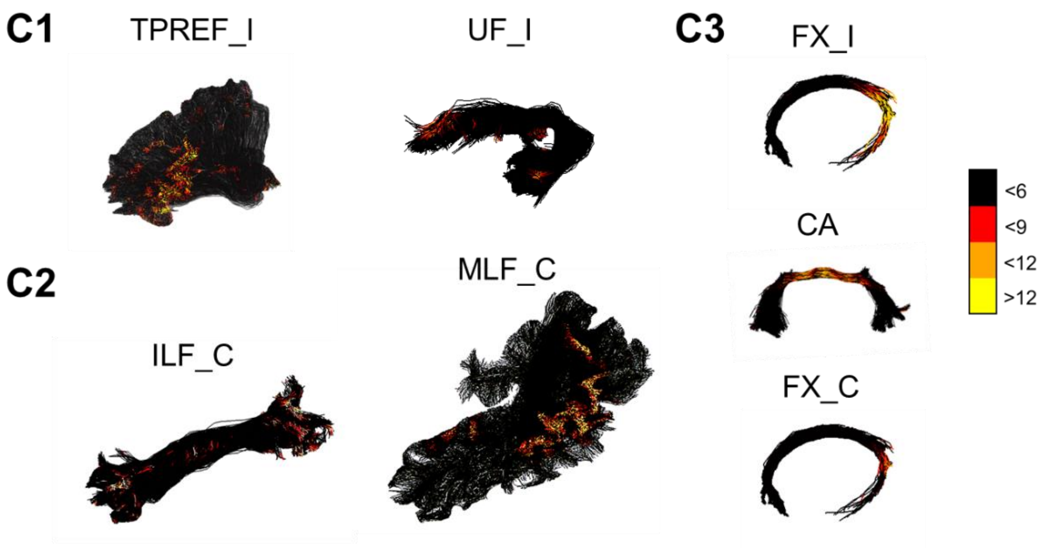

Figure S5: TWI-FA “abnormality prevalence maps” (tract level)

Spatial distribution map of TWI-FA lesions and their prevalence in patients. Prevalence was estimated at the FOI level from individual TWI-FA z-score maps (binarized on the basis of significant lesions; z < -4.8). Significantly altered voxels for: less than one-third of TLE patients are projected in black (n < 6); less than half of patients in red (n < 9); less than two-thirds of patients in orange (n < 12); and for more than two-thirds of patients in yellow (n > 12). Interestingly, there is some spatial homogeneity of the impairments between subjects, forming “portions” of altered fibers. Only FOIs with minimal agreement between subjects (i.e., at least 10% of injured voxels matching in more than 1/3 of the TLE) are shown here. These WM bundles originate primarily from (or connect) the temporal lobes. The spatial distribution of lesions on the other FOIs is variable between subjects (high heterogeneity). C1 = cluster 1 of TWI-FA damage (in terms of severity, see main text); C2 = cluster 2; and C3 = cluster 3.

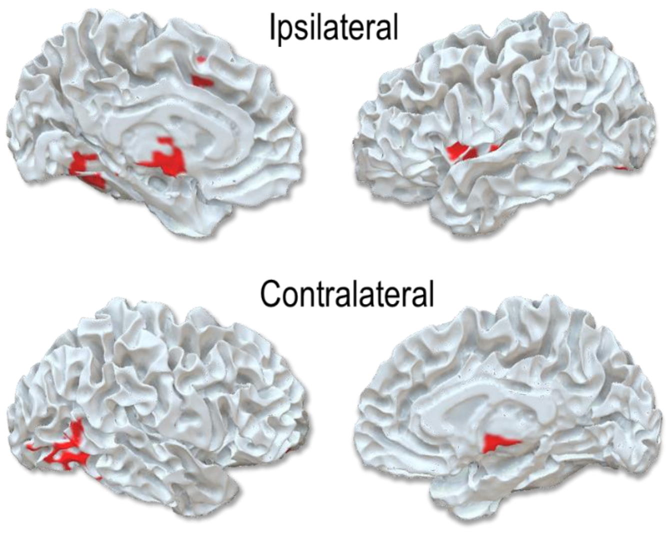

Figure S5: Consistency of the spatial distribution of TWI-FA anomaly (whole brain)

Cooccurrence of TWI-FA anomalies identified in TLE patients *versus* controls (whole brain lesion maps; all patients). Voxels altered in half of the patients (or more) were defined as “consistently impaired voxels” and are projected in red. They are mainly located in the temporal lobe, for both hemispheres, with however a predominance in the hemisphere ipsilateral to the epileptogenic region. Frontal damage was more variable between subjects. The lesion concordance index [i.e., the global ratio between the number of “consistently impaired voxels” and the number of significantly altered voxels (at the group level; corrected z scores < -4.8)] is 3476/11242 = 30.92%.

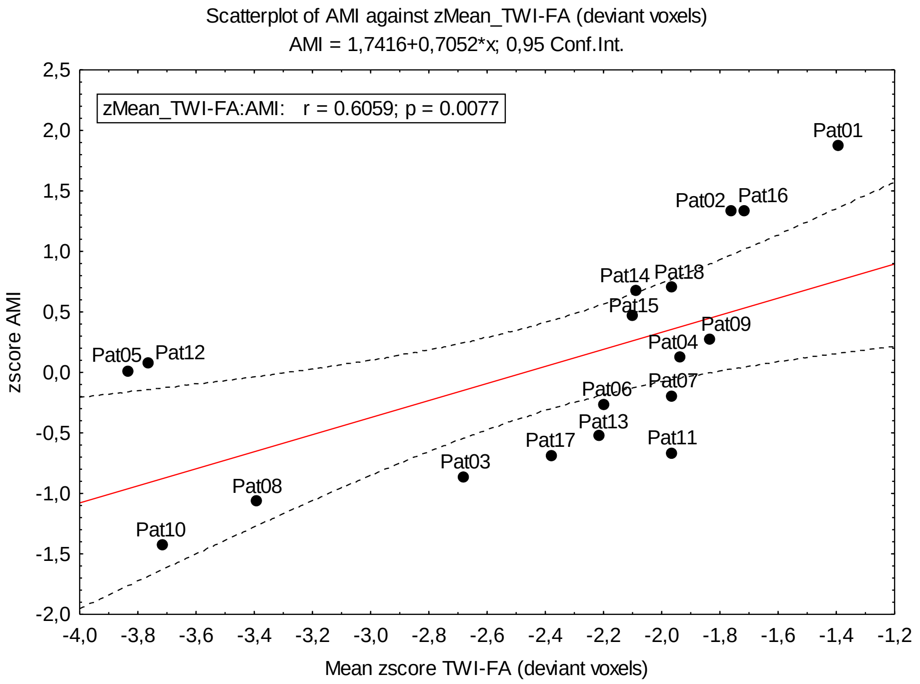

Figure S7: Scatterplot of the correlation observed between TWI-FA and AMI in TLE patients

We found a positive association (r = 0.61, p <.05) between mean TWI-FA z scores (estimated from voxels that diverge significantly in global manifold space; Figure 3 Panel B) and AMI cognitive z scores (Auditory Memory Index of the WMS IV: Weschler, 2012; Appendix S1). The graph shows the corresponding regression line (red line) and the 95% confidence interval (dotted lines).

Table S2: Correlation values (p < .05) between FOIs and clinical or cognitive variables

Cluster1			Cluster2			Cluster3		
**Source**	**Target**	**Value**	**Source**	**Target**	**Value**	**Source**	**Target**	**Value**
UF_C	AED	0.38281	CG_I	TMT	0.511526	CA	FRQ	0.506294
UF_I	AED	0.474736	ST_OCC_C	TMT	0.482666	FX_C	PRI	0.453334
ST_PREF_I	AED	0.479986	CC_6	TMT	0.40437	FX_C	ASY	0.545766
ST_FO_I	AMI	0.440075	OR_C	TMT	0.402592	FX_I	ASY	0.562149
T_PREF_I	AMI	0.435325	T_OCC_C	TMT	0.406287	FX_I	AMI	0.461907
UF_I	AMI	0.501114	ST_POSTC_I	TMT	0.541982	FX_I	NAM	0.470339
T_PREM_I	AMI	0.585083	FPT_I	TMT	0.49839	FX_I	HIP	0.655538
ATR_I	AMI	0.456129	IFO_C	VMI	0.419342			
ST_PREF_I	AMI	0.369336	MLF_C	NAM	0.584678			
T_PREF_I	ASO	0.519884	MLF_C	SFL	0.686458			
ATR_I	ASO	0.402125	FPT_I	ASO	0.409472			
ST_FO_I	EDU	0.445308						
CC_1	EDU	0.433119						
UF_I	EDU	0.401085						
ATR_I	EDU	0.445063						
ST_PREF_I	PFL	0.350462						
CC_1	PFL	0.398392						
CC_2	PFL	0.398392						

Table S3: Differences between controls and patients on TWI-AD and TWI-RD values for the FOIs of cluster 3

**FOIs Cluster 3**	T-tests; Grouping: Group 1: HC Group 2: TLE									
	Mean HC	Mean TLE	t-value	df	p	Valid N HC	Valid N TLE	Std.Dev. HC	Std.Dev. TLE	F-ratio Variances	p Variances
**TWI-RD_CA**	0.000621	0.000681	-2.20125	53	0.032095	37	18	0.000107	0.000061	3.044985	0.016818
**TWI-RD_FX_I**	0.000674	0.000800	-3.56458	53	0.000781	37	18	0.000111	0.000145	1.721711	0.168197
**TWI-RD_FX_C**	0.000660	0.000775	-2.78332	53	0.007441	37	18	0.000142	0.000149	1.108676	0.766538
**TWI-AD_CA**	0.001138	0.001054	5.17245	53	0.000004	37	18	0.000067	0.000028	5.647403	0.000387
**TWI-AD_FX_I**	0.002207	0.001853	4.73659	53	0.000017	37	18	0.000305	0.000121	6.331988	0.000178
**TWI-AD_FX_C**	0.002129	0.002188	-0.62461	53	0.534905	37	18	0.000301	0.000372	1.527617	0.279929

RD = radial diffusivity; AD = axial diffusivity; CA = anterior commissure; FX_I = ipsilateral fornix; FX_C = contralateral fornix. Significant differences between patients and healthy controls ($p < .05$) are marked in red. A negative t-value indicates an increase in the parameter in patients compared to controls. A positive t-value indicates a decrease of the parameter in patients compared to controls. In line with Tromp (2016), for example, patterns of significant decrease/increase in TWI-AD and TWI-RD in patients could be interpreted as an “axonal degeneration” for FX_I and for CA; and rather a “demyelination” for FX_C.

Reference

Tromp, D. (2016). DTI Scalars (FA, MD, AD, RD) - How do they relate to brain structure? The Winnower. https://doi.org/10.15200/winn.146119.94778
